# Transgenic Mice for a Tamoxifen-Induced, Conditional Expression of the Cre Recombinase in Osteoclasts

**DOI:** 10.1371/journal.pone.0037592

**Published:** 2012-05-18

**Authors:** Maria Arantzazu Sanchez-Fernandez, Silvia Sbacchi, Miguel Correa-Tapia, Ronald Naumann, Jennifer Klemm, Pierre Chambon, Samiya Al-Robaiy, Manfred Blessing, Bernard Hoflack

**Affiliations:** 1 Biotechnology Center, Dresden University of Technology, Tatzberg, Dresden, Germany; 2 Max Planck Institut for molecular cell biology and genetics, Pfotenhauerstrasse, Dresden, Germany; 3 Institut de Génétique et de Biologie Moléculaire et Cellulaire, CNRS UMR7104, Inserm U964, Université de Strasbourg, Collège de France, Illkirch, France; 4 Biotechnologish-Biomedizinisches zentrum, Deutscher Platz 5, Leipzig Germany; Sanford Burnham Medical Research Institute, United States of America

## Abstract

**Background:**

Studies on osteoclasts, the bone resorbing cells, have remained limited due to the lack of transgenic mice allowing the conditional knockout of genes in osteoclasts at any time during development or adulthood.

**Methodology/Principal Finding:**

We report here on the generation of transgenic mice which specifically express a tamoxifen-inducible Cre recombinase in osteoclasts. These mice, generated on C57BL/6 and FVB background, express a fusion Cre recombinase-ERT2 protein whose expression is driven by the promoter of cathepsin K (CtsK), a gene highly expressed in osteoclasts. We tested the cellular specificity of Cre activity in CtsKCreERT2 strains by breeding with Rosa26LacZ reporter mice. PCR and histological analyses of the CtsKCreERT2LacZ positive adult mice and E17.5 embryos show that Cre activity is restricted largely to bone tissue. *In vitro*, primary osteoclasts derived from the bone marrow of CtsKCreERT2+/−LacZ+/− adult mice show a Cre-dependent β-galactosidase activity after tamoxifen stimulation.

**Conclusions/Significance:**

We have generated transgenic lines that enable the tamoxifen-induced, conditional deletion of loxP-flanked genes in osteoclasts, thus circumventing embryonic and postnatal gene lethality and avoiding gene deletion in other cell types. Such CtsKCreERT2 mice provide a convenient tool to study *in vivo* the different facets of osteoclast function in bone physiology during different developmental stages and adulthood of mice.

## Introduction

The bone tissue is continuously remodelled throughout life to maintain its physical properties. This remodeling process, in which the bone matrix is digested and rebuilt, requires active bone resorbing osteoclasts and bone rebuilding osteoblasts. The activity of these two cell types is tightly coupled so that bone degradation is balanced by bone formation during aldulthood [1], [2], [3]. Many skeletal diseases result from a loss of this balance, as exemplified by osteoporosis, a disease in which the activity of osteoclasts overcomes the activity of osteoblasts leading to a decrease of the bone mass [4].

Many studies have illustrated how osteoblasts control the differentiation of osteoclast precursors into mature polynucleated cells and thus bone degradation [5]. Osteoblasts and stromal cells express the macrophage stimulating colony factor (MCSF), which controls osteoclast precursor proliferation and the Receptor for Activation of NF-kB Ligand (RANKL), which controls their differentiation. There is evidence that osteclasts can control osteoblast differentiation and thus bone rebuilding, as illustrated by Ephrin E2 expressed by osteoclasts and recognized by Ephrin B4 receptor expressed by osteoblasts, thereby modulating osteogenic differentiation. [6], [7], [8]. Mature osteoclasts also secrete growth factors such as PDGF-BB, which is recognized by PDGF receptor beta on osteoblasts and their precursors and stimulate their chemotaxis [9].

Studies on osteoclasts have been hampered by the lack of proper systems to explore, both *in vitro* and *in vivo*, their function in bone digestion and their relationships with other cell types that contribute to bone homeostasis. Osteoclastogenesis can now be recapitulated *in vitro* by stimulating osteoclast precursors with recombinant MCSF and RANKL, thus facilitating biochemical and cell biological studies. However, the systems to specifically inactivate genes in osteoclasts have not been totally exploited. Transgenic mouse lines that express the Cre recombinase, whose expression is driven by the promoters of cathepsin K (CtsK) or tartrate-resistent acid phosphatase (TRAcP), two lysosomal hydrolases highly expressed in osteoclasts, have been generated [10]. If such mouse lines enable cell/tissue specific deletion of loxP site-flanked target genes in mouse osteoclasts, they do not overcome embryonic or perinatal lethality and do not allow to precisely manipulate the timing of recombination, especially to study gene function during adulthood. The Cre recombinase fused to a mutant ligand-binding domain of the estrogen receptor (CreERT2) circumvents this problem [11], [12]. CreERT2 has high affinity for the 4-hydroxytamoxifen (4-OHT) but not for the endogenous estradiol. After its exposure to the specific inducer, CreERT2 is translocated into the nucleus and mediates the recombination of floxed genes. This allows the control of the activation/inactivation of specific genes during a precise stage of embryogenesis and aldulthood [13].

We report here on the generation of transgenic CreERT2 mouse lines, in which the cathepsin K promoter drives CreERT2 expression. Cathepsin K is a cysteine protease drastically upregulated during osteoclastogenesis and is commonly used as an osteoclast marker [10], [14]. The characterization of these mouse lines indicates that CreERT2 is essentially functional in osteoclasts. These CtsKCreERT2 transgenic mouse lines provide a suitable tool to specifically inactivate any loxP-targeted gene in osteoclasts in a spatial and temporal manner.

## Results and Discussion

### Targeting construct and validation in cells

**Figure 1:**
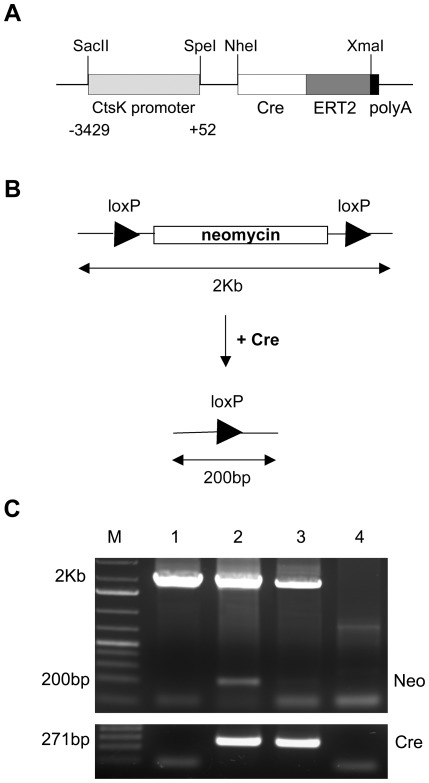
CtsKCreERT2 construct and *In vitro* functionality. **A**: Schematic diagram of transgenic CtsKCreERT2 expression construct used to generate transgenic mice, which includes the full sequence of the CtsK promoter, the CreERT2 fusion sequence, and the SV40 polyA signal. **B.** Schematic representation of the floxed neomycin construct before and after Cre-mediated recombination and expected size of PCR products. **C**: Cre-mediated recombination in Hela cells (upper panel). Hela cells transfected with the floxed neomycin plasmid only (lane 1) or co-transfected with CtsKCreERT2 and floxed nemycin constructs and treated (lane 2) or not (lane 3) with 4-OHT. Untrasfected Hela cells are shown in lane 4. The recombination resulted in a 200 bp PCR fragment. Cre expression was also monitored in the same samples by PCR (lower panel). M: molecular weight markers.

To generate an inducible osteoclast specific Cre transgenic mouse line, we first cloned the mouse cathepsin K promoter (−3429 to +52 from the start codon). This fragment was inserted upstream of a tamoxifen-regulated Cre recombinase-ERT2 plasmid (Fig. 1A). In order to test the functionality of this tamoxifen inducible CtsKCreERT2 construct *in vitro*, Hela cells were co-transfected with the CtsKCreERT2 construct and a plasmid containing a 2 kb long, *loxP*-flanked neomycin cassette. Cells were then treated with 4-OHT and the functionality of the CreERT2 transgene was measured by the loss of the *loxP* flanked neomycin cassette (Fig. 1B), resulting in appearance of a 200 bp PCR fragment (Fig. 1C). This promoter region was sensitive to RANKL induction when expressed in the Raw264.7 macrophage cell line (data not shown). This CtsKCreERT2 construct was then used to generate transgenic mice.

### Generation of transgenic mice and characterization

**Figure 2:**
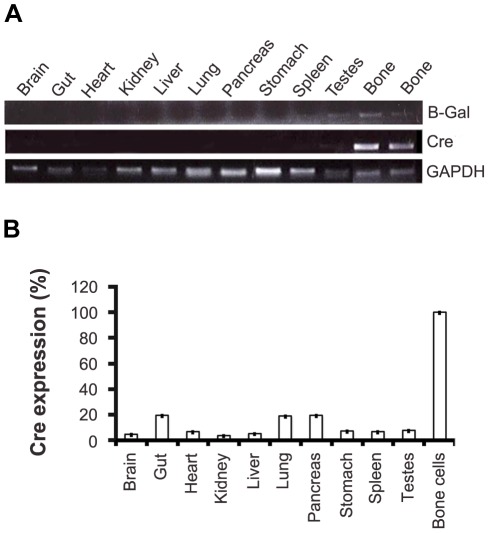
Expression of Cre and β-galactosidase in adult mouse tissues. β-galactosidase and Cre expression in organs and bones of CtsKCreERT2+/−LacZ+/− (strain #4).Total RNAs were isolated from various tissues and expression was detected by semi quantitative (A) and quantitative (B) PCR as described in materials and methods. GAPDH was used as internal control and normalization.

The CtsKCreERT2 construct was injected into oocytes of C57BL/6 and FVB mice. Out of fifty mice, seven C57BL/6 and two FVB conditional Cre positive strains were identified as potential founders and mated with C57BL/6 mice. The FVB founders were crossed for more than eight generations with C57BL/6 mice to eliminate their FVB background. Our preliminary PCR characterization showed that CreERT2 mRNAs could be detected in bones but not significantly in other tissues (data not shown) and therefore every strain appeared suitable for further characterization. One mouse founder from each background was selected for further characterization. To define the ability of the CreERT2 to induce specific recombination in the bone tissue, offsprings of the two selected CreERT2 expressing transgenic lines, one originating from a founder with a C57BL/6background (strain #4) and one originating from a founder with a FVB background (strain #284), were bred with the ROSA26loxLacZ reporter strain (R26R), in which the ROSA26 locus has been targeted with a *loxP* flanked stop codon cassette and the *LacZ* gene encoding β-galactosidase [15]. Offspring (8–10 weeks old) from crosses between mice haboring the R26R locus and CtskCreERT2 transgenic mouse lines were treated with 4-hydroxytamoxifen. Cre recombinase and β-galactosidase expression was triggered by the Cre-mediated deletion of the loxed stop codon cassette, which activated transcription of the *LacZ* gene. Cre and LacZ activities were monitored by semi-quantitative PCR. Figure 2A shows that the Cre recombinase and β-galactosidase could be easily detected in bones (strain # 4) but not in other tissues with the exception of testes. Semi quantitative RT-PCR performed on tissues (Fig. 2B) also revealed some residual Cre expression in other tissues like lungs and pancreas. However, β-galactosidase activity could not be detected by histochemistry in these tissues or others as illustrated in Fig. S1.

### Characterization of Cre-ERT2 mouse embryos

**Figure 3:**
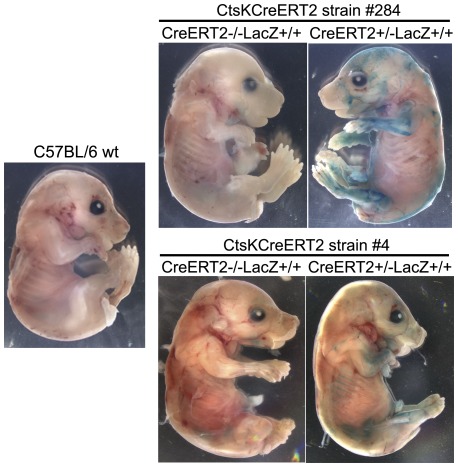
Tissue distribution of functional Cre recombinase in CtsKCreERT2LacZ E17.5 embryos. CtsKCreERT2LacZ and wild-type females were mated with ROSA26 males. Pregnant females were treated with tamoxifen as described in materials and methods. At day E17.5, the embryos were collected and analyzed for β-galactosidase activity (X-gal staining) as described in materials and methods using embryos with various genotypes.

**Figure 4:**
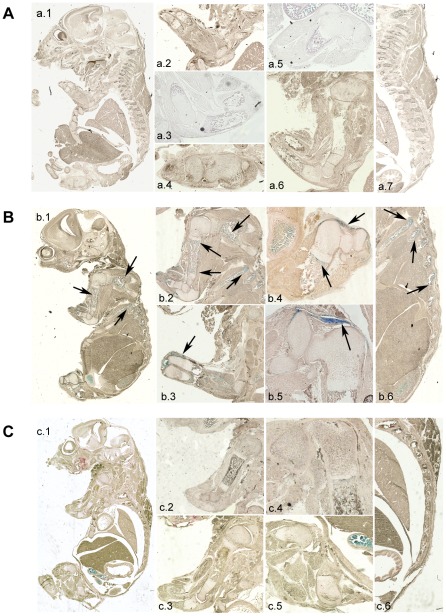
Cre-mediated β-galactosidase expression in tissue sections of transgenic embryos. E17.5 embryos obtained after crossing CtsKCreERT2 positive or wild-type females with ROSA26 males were processed and stained for β-galactosidase activity as described under materials and methods. **A**: E17.5 C57BL/6 wild-type control embryos; **a.1**: overview of β-gal activity; **a.2**, **a.3** and **a.6**: arms; **a.4** and **a.5**: legs; **a.7**: spinal cord **B**: E17.5 CtsKCreERT2+/−LacZ+/+ embryos (strain #4); **b.1**: overview of β-gal activity. **b.2** and **b.3**: arms; **b.4** and **b.5** legs; **b.6**: spinal cord. Arrows show positive β-galactosidase activity in bones. **C**: E17.5 CtsKCreERT2−/−LacZ+/+ embryos (strain #4); **c.1**: overview of β-gal activity; **c.2** and **c.3**: arms; **c.4** and **c.5**: legs; **c.6**: spinal cord.

**Figure 5:**
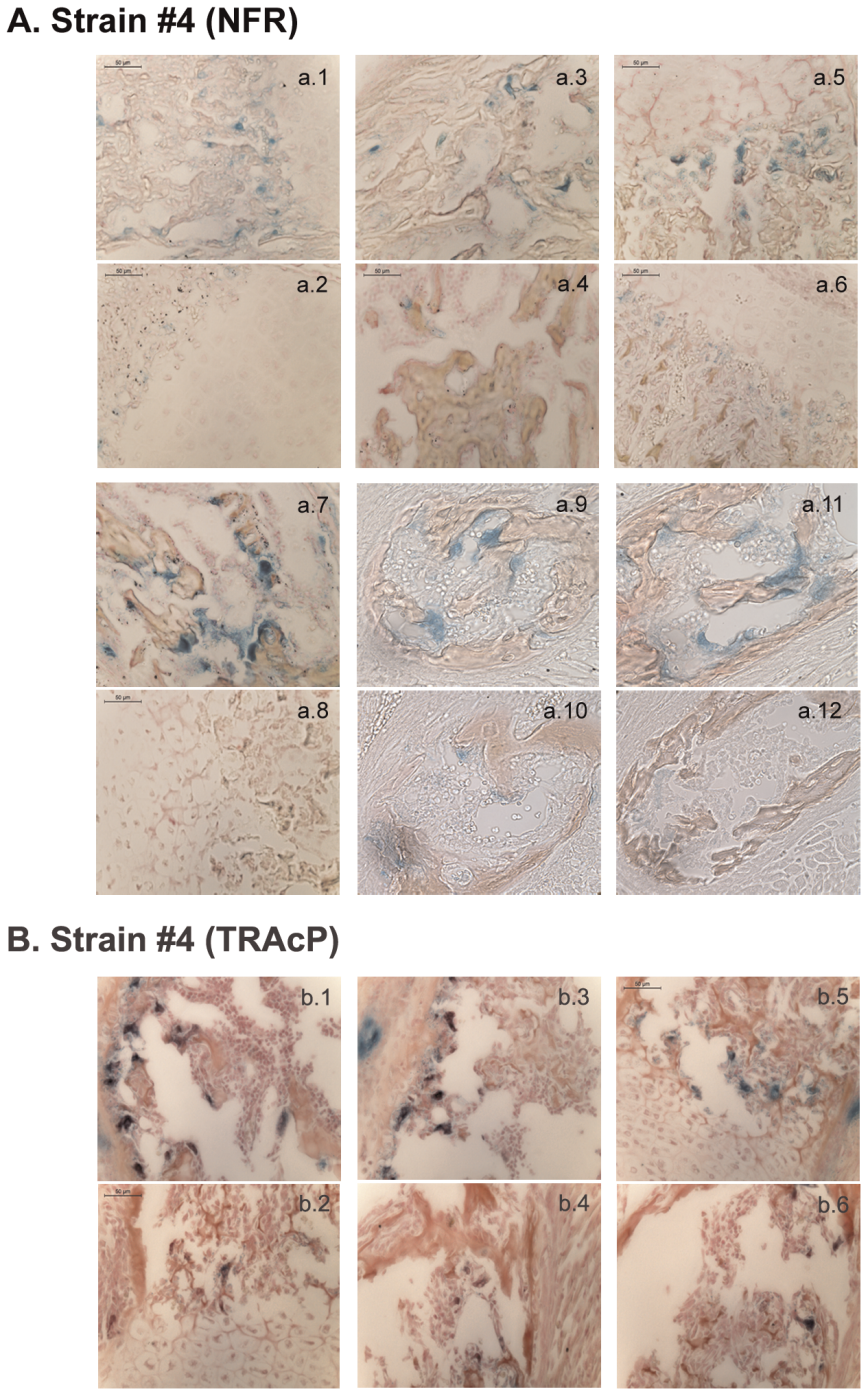
Detailed Cre-mediated β-galactosidase expression in tissue sections of transgenic embryos. 17.5-day-old embryos with different genotypes (CreERT2+/−LacZ+/+ and CreERT2−/−LacZ+/+; strain #4) were collected after 3 days of tamoxifen induction and processed for histochemistry to detect β-galactosidase activity. Sections were either co-stained with Nuclear Fast Red (NFR) (**A**) or Tartrate-resistent acid phosphatase (TRAcP) (**B**). **a.1** and **a.3**: legs of CtsKCreERT2+/−LacZ+/+ embryos; **a.2** and **a.4:** legs of CtsKCreERT2−/−LacZ+/+ embryos; **a.5** and **a.7:** arms of CtsKCreERT2+/−LacZ+/− embryos; **a.6** and **a.8:** arms of CtsKCreERT2−/−LacZ+/+ embryos; **a.9** and **a.11:** vetebraes of CtsKCreERT2+/−LacZ+/− embryos; **a.10** and **a.12:** vetebraes of CtsKCreERT2−/−LacZ+/+ embryos. **b.1, b.3** and **b.5:** arms of CtsKCreERT2+/−LacZ+/+ embryos; **b.2, b.4** and **b.6:** arms of CtsKCreERT2−/−LacZ+/+ embryos.

We next investigated the specificity of recombination in embryos. CtsKCreERT2LacZ positive females were crossed with R26R males. Pregnant females were fed with tamoxifen for 3 days at day E13.5 of embryogenesis. Embryos were collected at day E17.5 and then processed to detect β-galactosidase activity in tissues. Embryos from CtsKCreERT2 lines #4 and #284 showed intense staining in long bones. Staining could also be seen in vertebrae, calvaria and craniums (Fig. 3). As expected, no staining was observed in CtsKCreERT2−/−LacZ+/+ or C57BL/6 wild-type embryos. These results strongly suggest that the tissue-specific activation of the Cre recombinase had occurred in mouse line #4 and #284. To observe in more details the specificity of Cre recombination, paraffin sections of the β-galactosidase-stained embryos were prepared. Sections of strain #4 embryos showed an intense β-galactosidase activity in long bones (Fig. 4B; b.1, b.2 and b.4,), vertebrae (Fig. 4B; b.1 and b.6), ribs (Fig. 4B; b.2 and b.6), cranium and calvaria (Fig. 4B; b1). In contrast, CtsKCreERT2−/−LacZ+/− and C57BL/6 wild-type embryos did not exhibit any β-galactosidase activity in any tissue including bones (Fig. 4A and C), with the exception of the intestine as usually observed in all embryos. Sections of strain #284 embryos showed similar results (data not shown). It is worth noting that some β-galactosidase staining was observed at junctions between femur and tibia of embryos (Fig. 4B, b.4, b.5) as well as in the periostium (Fig. 4B, b.3; Fig. 5B, b.3). These mononucleated cells were not positive for the osteoclast marker TRAcP (Fig. 5B; b.3 left). Because joints, bone and its periostium are high vascularized, these cells could represent mononucleated osteoclast precursors already committed to osteoclastic differentiation [16]. At higher magnifications, β-galactosidase-positive, mutinucleated osteoclasts could clearly be observed in CreERT2+/−LacZ+/+ long bones, especially in the areas where osteoclasts are usually observed (Fig. 5A; a.1, a.3, a.5 and a.7), and in vertebrae (Fig. 5A; a.9 and a.11). These osteoclasts were also TRAcP positive (Fig. 5B).

### Cre-ERT2 activity in primary osteoclasts differentiated *in vitro* from bone marrow

**Figure 6:**
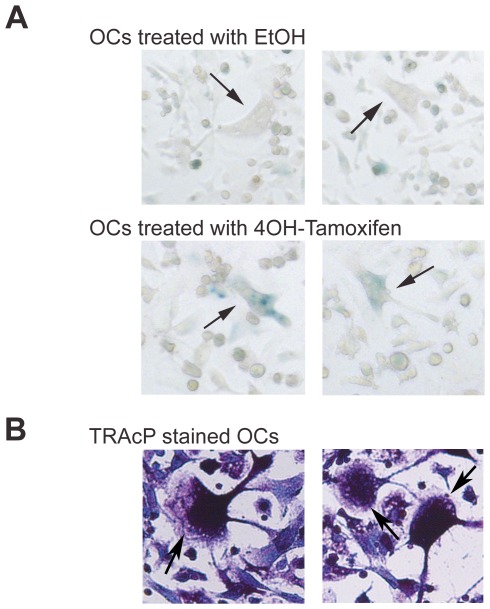
Cre-mediated β-Galactosidase activity in osteoclasts derived from bone marrow of CtsKCreERT2/LacZ-positive transgenic mice. Primary osteoclasts were differentiated from bone marrow of tibias and femurs of CtsKCreERT2+/−LacZ+/− strain #4 as described in materials and methods. They were then treated with 1 µM 4-OHT or EtOH alone. After 24 hours, they were then treated to detect their β-galactosidase activity (A) or their TRAcP activity (B). The arrows indicate multinucleated osteoclasts.

Primary osteoclasts were differentiated and induced with 4-OHT to test the functionality of the CreERT2 transgene *in vitro*. Bone marrow from 8–10 weeks old CtsKCreERT2+/−LacZ+/− mice (strain #4) was extracted. Bone marrow cells were treated with MCSF and RANKL to induce osteoclast differentiation. The differentiated cells were then treated with tamoxifen, and the β-galactosidase expression was tested by detecting its enzyme activity. Although some endogenous β-galactosidase staining was detected in mononucleated cells treated only with vehicle (96%EtOH) [17], [18], primary multinucleated osteoclasts of the transgenic mouse strain #4 showed a more intense β-galactosidase staining than negative controls (Fig. 6A). These multinucleated cells were also positive for TRAcP activity (Fig. 6B)

Bone research has been facilitated by the generation of transgenic mice expressing the Cre recombinase under the control of TRAcP or CtsK promoters [10]. This latter study showed that the CtsK promoter is more appropriate to specifically inactivate genes in osteoclasts than the TRAcP promoter. Cathepsin K is not only expressed in osteoclasts but also in other cell types, as evidenced by a detailed analysis of cathepsin K-deficient mice [19], [20], [21]. However, cathepsin K is drastically overexpressed during RANKL-induced osteoclastogenesis (a 200–400 fold increase in expression). This certainly allows a more efficient expression of CreCRT2 in osteoclasts than in other tissues. The mouse lines described in our study were selected for expressing detectable levels of CreERT2 in osteoclasts and no detectable levels of CreERT2 in other tissues expressing cathepsin K although some leakiness was still observed. Thus, these conditional CtsKCreERT2 mice may provide an additional convenient tool to illustrate the physiological importance of genes essential for osteoclast function during development and, in particular, during adulthood when the activity of osteoclasts in bone degradation becomes more prominent and cannot be compensated by the activity of osteoblasts in bone rebuilding, as seen in osteoporosis.

## Materials and Methods

### Generation of CtsKCreERT2 transgene

The CtsKCreERT2 construct was generated by including the mouse CtsK gene sequence (−3429 to +52) into a CreERT2 plasmid. Standard restriction digestions (SacII and SpeI) and cloning techniques were used.

### Testing the CtsKCreERT2 transgene *in vitro*

To assess the functionality of CreERT2 protein *in vitro*, an equimolar ratio of CtsKCreERT2 construct and a plasmid containing the selectable marker neomycin flanked by *loxP* sites were transiently cotransfected in Hela cells. After 24 hours, the transfected cells were treated with 1 µM tamoxifen during 24 hours. Then, the HeLa cells were harvested and the genomic DNA was extracted. Cre-mediated recombination was determined by PCR using the primers flanking the *loxP* sites: F: 5′-CGACAACCACTACCTGAGCA-3′ and R: 5′-GGATGCAGAGTGTGCGTAGA-3′.

### Generation and identification of CtsKCreERT2 transgenic mouse lines

A 1,7 kb fragment of the original vector was produced by XmaI-SacII digestion. This fragment was microinjected into the pronuclei of C57BL/6 or FVB fertilized mouse eggs and transferred to pseudo-pregnant females. Founder mice were genotyped using tail genomic DNA (gDNA) by polymerase chain reaction (PCR) with the Cre-specific primers, F: 5′-CGGTCTGGCAGTAAAAACTAT-3′ and R: 5′-CAGGGTGTTATAAGCAATCCC-3′. Genomic DNA was extracted from tails using the Archivepure DNA Mouse Tail kit (5PRIME, Germany). The Cre founders were back-crossed into the C57BL/6 background for production of transgenic offsprings. Animals were housed at the Biomedical Service Facility (BMS) of the MPI-CBG institute Dresden) at 24°C with a 12 hours light-dark cycle and tap water as well as food were supplied ad libitum.

### RNA expression levels of Cre recombinase in CtsKCreERT2 transgenic mouse line #4

Positive animals between 8 and 10 weeks of age were sacrified to isolate long bones (femur and tibia) and other organs. Tibias and femurs were cleaned of the surrounding soft tissue. Following excision of the ends of the long bones, bone marrow was removed by flushing with ice-cold PBS. The remaining bone was crushed with sterile forceps and homogenized (Polytron) in RNase inhibiting solution (4 M guanidine isothiocyanate, 25 mM sodium citrate, pH 7.0, 0.5% sarcosyl, 0.1 M 2-mercaptoethanol). The homogenates were stored at −80°C prior to total RNA extraction. Total RNA extraction was based on the acid guanidinium thiocyanate-phenol-chloroform single-step method [22]. A 50 mg piece of each collected organs was homogenized (Polytron) in 1 ml TRIZOL reagent. Total RNA was then isolated with chloroform/isopropanol using peqGOLD phase trap tubes (PEQLAB Biotechnologies GmbH, 91052 Erlangen, Germany). Quantitative RT-PCR (QPCR) using the above Cre-specific primers determined the tissue distribution of Cre recombinase expression. QPCR was performed with an Agilent Technologies Mx3000P system and the Brilliant II SYBR Green QPCR kit according to manufacturer's instructions (Agilent Technologies, Böblingen, Germany). Quantitative RT-PCR analyses were performed in triplicates, and GAPDH (F: 5′-TCACCACCATGGAGAAGGC-3′and R: 5′-GCTAAGCAGTTGGTGGTGCA-3′) was used to normalize the data.

### Cre recombinase functionality in embryos

To analyze the functionality and the distribution of the Cre recombinase in embryos, CtsKCreERT2LacZ females were crossed with ROSA26 reporter (R26R) males [15]. Pregnant females were oral fed with 4 mg of 4-OHT for 3 consecutive days starting at 13.5 dpc. Pregnant mice were sacrificed 48 hours after the last oral feeding, and the embryos were isolated and processed for whole-mount X-gal staining. Briefly, skin was removed and embryos were fixed for 1 hour at RT with 0.2% glutaraldehyde, 2% formaldehyde, 2 mM MgCl2 in PBS (pH 7.3). Embryos were then rinsed three times for 30 minutes in 0,1 M PBS (pH 7.3) containing 2 mM MgCl2, 0.02% (vol/vol) Nonidet P-40, 0.01% sodium deoxycholate. Staining was performed over night at 37°C with the same rinsing solution supplemented with 1 mg/ml of 5-bromo-4-chloro-3-indolyl β-D-galactopyranoside (X-gal), 5 mM potassium ferrocyanide and 5 mM potassium ferricyanide. Color development was stopped by immersion of samples in PBS (pH 7.8) with 10 mM EDTA (pH 8.0). Pictures of the samples were taken with the Zeiss SteMi Discovery V20 microscope.

For histological analysis of the X-gal stained embryos, samples were postfixed in 2% formaldehyde (pH 7.8) overnight at 4°C. Then, the embryos were dehydrated and paraffin embedded. Sections of 8 µm were cut and counterstained with nuclear fast red (NFR) (Sigma-Aldrich). Pictures of embryo sections were taken with a Zeiss – Upright motorized Apotome. For more detailed pictures of osteoclasts in embryo sections the Leica upright videomicroscope was used. TRAcP staining, to localize osteoclasts, was also performed in some of the embryo sections using a TRAcP staining kit and according to manufacturer's instructions (Sigma-Aldrich).

### Cre recombinase functionality in tissues of adult mice

To analyse β-galactosidase expression in tissues of adult mice, 8-week-old wild-type C57BL/6 and CtsKCreERT2+/−LacZ+/+ strain #4 mice were used. Mice were oral fed with 4 mg of 4-OHT for three days. After 48 hours, animals were perfused intracardially with PBS. Organs were then collected and total RNA isolated as described above. For histochemistry analises of β-galactosidase activity, mice were fix by 0.2% glutaraldehyde (GA) followed the PBS perfusion. Organs were then collected and postfixed in 0.2% GA for extra 4 hours. Organs were then cryopreserved in 30% sucrose/PBS overnight at 4°C and embedded in O.C.T. compound (Tissue-Tek®). Cryosections (8 µm) of each organ were made and stained for β-galactosidase activity. In short: cryosections were postfixed 10 minutes in 0.2% GA, washed three times 5 minutes in LacZ wash buffer (2 mM MgCl2, 0.02% NP-40, and 0.01% sodium deoxycholate in PBS) and incubated 3, 6 and 12 hours at 37°C in LacZ staining buffer (LacZ wash buffer supplemented with 1 mg/ml of 5-bromo-4-chloro-3-indolyl β-D-galactopyranoside (X-gal), 5 mM potassium ferrocyanide and 5 mM potassium ferricyanide). After incubation, sections were washed three times 5 minutes in PBS, fixed 10 minutes in 0.1% GA, washed three more times in PBS and counterstained with NFR. After a dehydratation with 50, 70, 100% EtOH and Xilol, organ sections were mounted with Cytoseal XYL (Richard-Allan Scientific). Pictures were taken with a WF Olympus upright, microscope.

### Cre recombinase functionality in osteoclasts

Bone marrow cells of 8 to 10 weeks old CtsKCreERT2+/−Lacz+/− mice femurs and tibias were flushed out with αMEM medium. Cells were then differentiated into osteoclasts or macrophages by respective addition of 20 ng/ml Macrophages Colony Stimulating Factor (MCSF, PeproTech, germany) and 50 ng/ml recombinant soluble Receptor activator of nuclear factor kappa-B ligand (RANKL, PeproTech, germany) or MCSF alone. Cells were grown in αMEM medium supplemented with 10% FCS, 2 mM HEPES, 1% penicillin/streptomycin and 1% L-glutamine in 10% CO_2_, 95% humidity at 37°C. After differentiation, 4-OHT (10^−3^ M) diluted in ethanol was added to the cultured cells. Ethanol alone was used for control cells. After 24 hours X-gal staining was performed using an *In Situ* β-galactosidase Staining Kit (Agilent Technologies, Böblingen, Germany) according to the manifaturer's instructions. Cells were stained for 6–8 hours at room temperature, washed three times with PBS and then observed under light microscopy (Zeiss Apotome microinjection videomicroscope, Germany).

## Supporting Information

Figure S1 **β-galactosidase activity in tissue sections of adult mice.** 8-week-old mice (CreERT2+/−LacZ+/− strain #4 and wild-type C57BL/6) were sacrificed after 3 days of tamoxifen induction. Organs were collected, cryosectioned (8 µm) and processed for histochemistry to detect β-galactosidase as describe in materials and methods. Organ cryosections were then counterstained with NFR. **A:** Thyroid glands sections of wild-type C57BL/6 and CtsKCreERT2 strain #4. **B:** Testis sections of wild-type C57BL/6 and CtsKCreERT2 strain #4. Arrows show β-galactosidase activity (in blue). **C:** Lung sections of wild-type C57BL/6 and CtsKCreERT2 strain #4. **D:** Pancreas sections of wild-type C57BL/6 and CtsKCreERT2 strain #4.
(TIF)
