# Supplementary figures and images for: Transgenic Mice for a Tamoxifen-Induced, Conditional Expression of the Cre Recombinase in Osteoclasts
Source: PLoS One. 2012 May 18;7(5):e37592. doi: 10.1371/journal.pone.0037592 (PMC3356310; doi:10.1371/journal.pone.0037592)

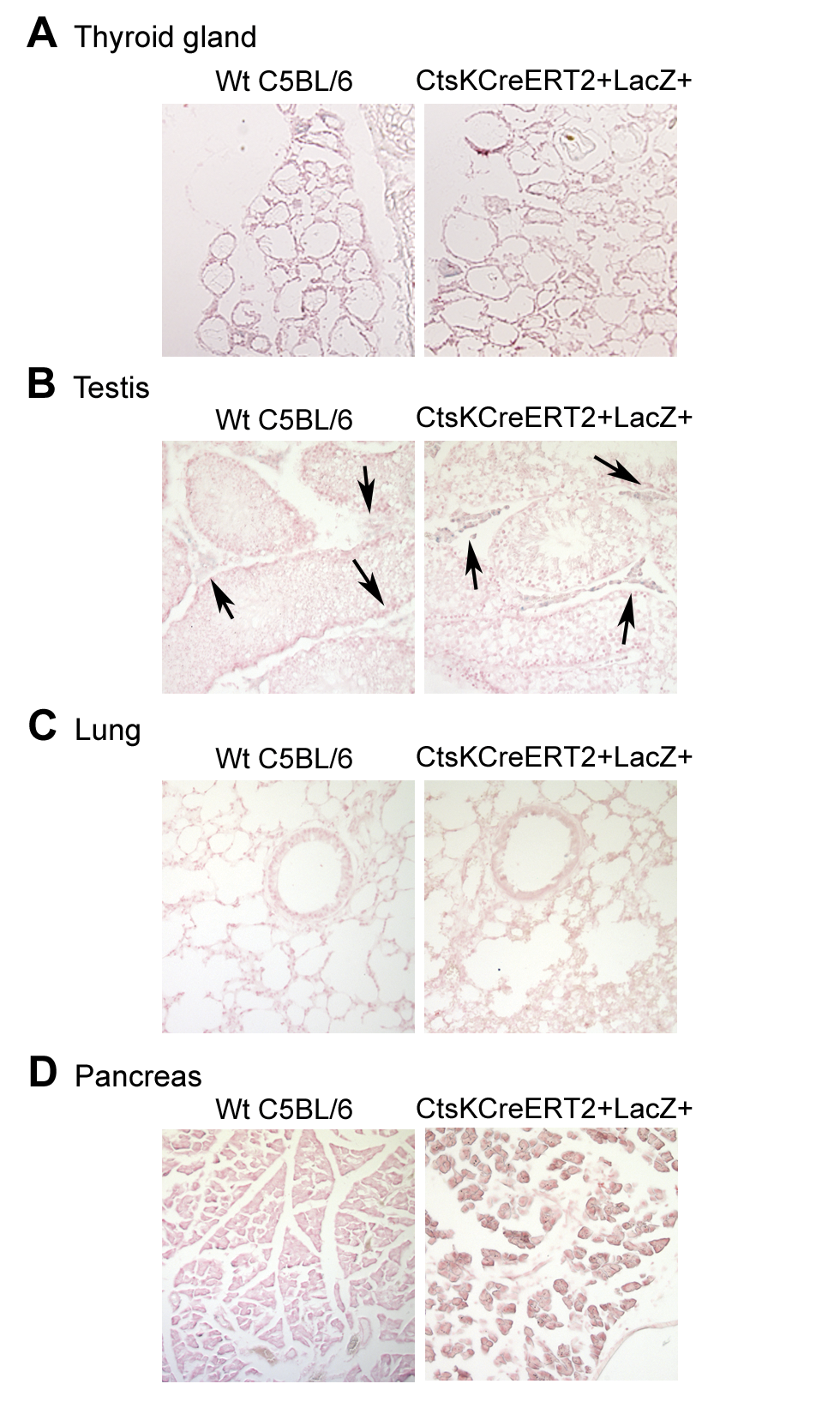

Supplement: Figure S1 — β-galactosidase activity in tissue sections of adult mice. 8-week-old mice (CreERT2+/−LacZ+/− strain #4 and wild-type C57BL/6) were sacrificed after 3 days of tamoxifen induction. Organs were collected, cryosectioned (8 µm) and processed for histochemistry to detect β-galactosidase as describe in materials and methods. Organ cryosections were then counterstained with NFR. A: Thyroid glands sections of wild-type C57BL/6 and CtsKCreERT2 strain #4. B: Testis sections of wild-type C57BL/6 and CtsKCreERT2 strain #4. Arrows show β-galactosidase activity (in blue). C: Lung sections of wild-type C57BL/6 and CtsKCreERT2 strain #4. D: Pancreas sections of wild-type C57BL/6 and CtsKCreERT2 strain #4. (TIF) [file pone.0037592.s001.tif]
